# Supplementary figures and images for: CRISPR/FnCas12a-mediated efficient multiplex and iterative genome editing in bacterial plant pathogens without donor DNA templates
Source: PLoS Pathog. 2023 Jan 10;19(1):e1010961. doi: 10.1371/journal.ppat.1010961 (PMC9870152; doi:10.1371/journal.ppat.1010961)

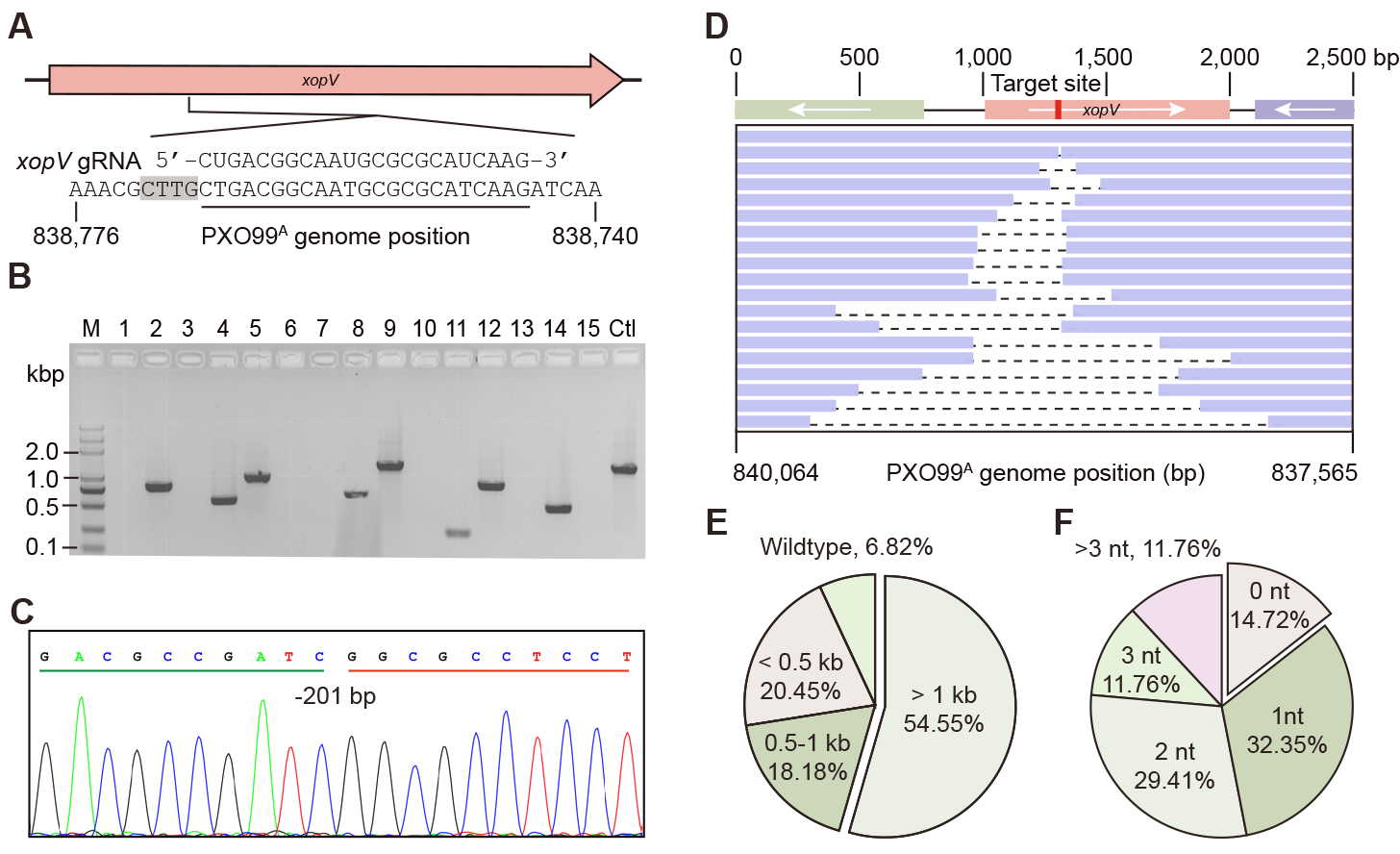

Supplement: S1 Fig — (A) The target site of xopV. The target region in the PXO99A genome is underlined and the PAM sequence is marked by the black shadow. (B) Single colonies were randomly selected for preliminary identification by PCR amplification of a 1.1 kb genomic fragment flanking the xopV gene. M, 5 kb DNA ladder; Ctl, control. (C) Representative Sanger sequencing chromatogram of the deletion mutant. -201bp, 201-bp deletion. (D) Deletion size distribution of mutants determined using tiling PCR and Sanger sequencing. The first line is wild type, other lines represent independent mutants, the dashed line in the middle indicates the deleted portion of each mutant, and the target site location is marked in red. (E) The pie chart represents the proportion of different bidirectional deletion ranges. (n = 44 individual colonies randomly selected). (F) As for (E), but depicting the ratio of the different flanking micro-homology regions used for DSB repair. (TIF) [file ppat.1010961.s001.tif]

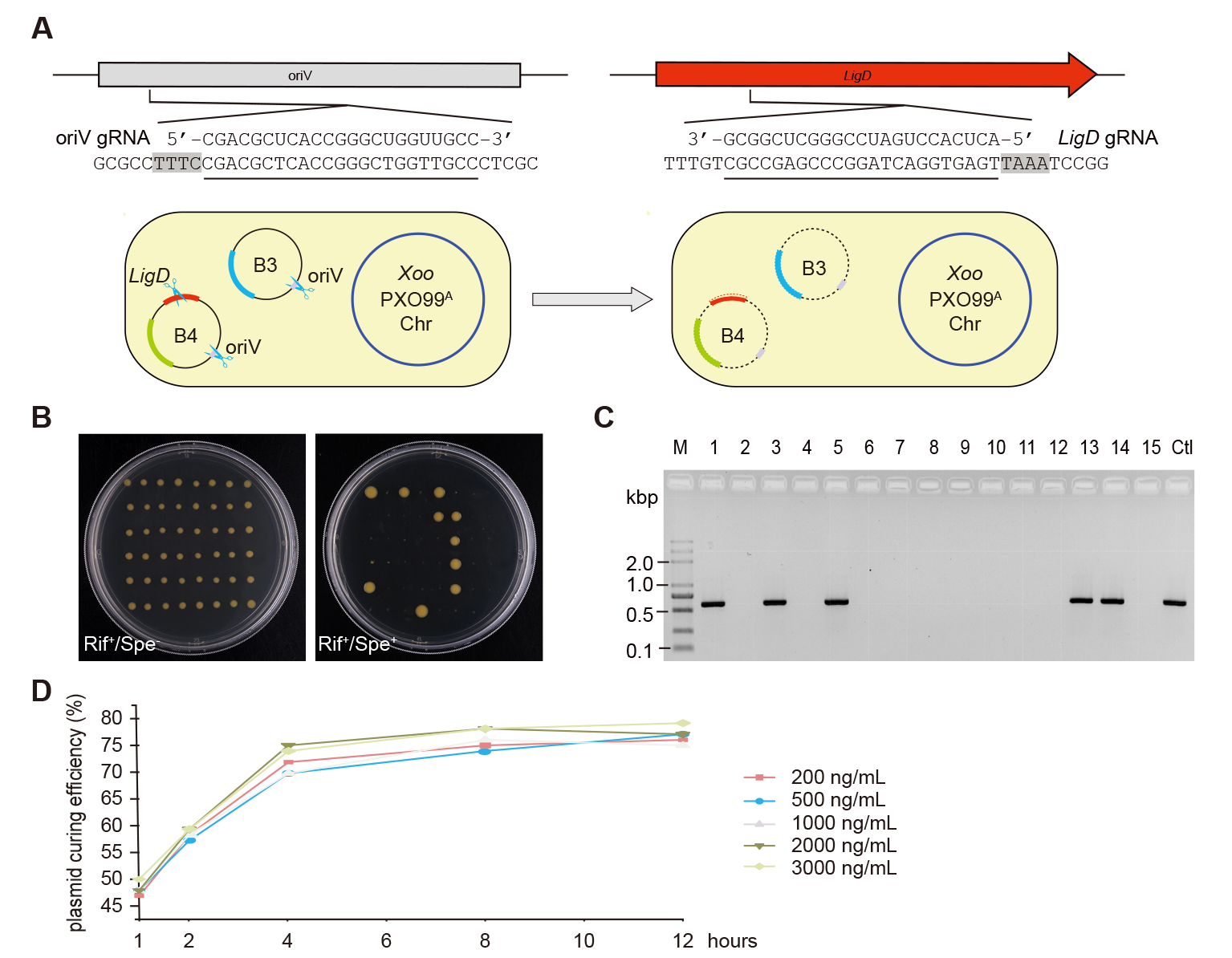

Supplement: S2 Fig — (A) CRISPR/FnCas12a nuclease-mediated cleavages of both oriV replicon and mtLigD gene resulted in the simultaneous removal of pHM1B3 and pHM1B4 plasmids. A crRNA array was designed to target the oriV replicon of pHM1 and the mtLigD gene in pHM1B4, respectively. The target regions in plasmids are underlined and the PAM sequences are marked by the black shadow. The expression cassettes of FnCas12a/xopN-crRNA, mtKu/mtLigD, and FnCas12a/oriV/mtLigD-crRNA are depicted as yellowgreen, red, and deepskyblue stripes. B3, pHM1B3-VD plasmid; B4, pHM1B4-crRNA plasmid. (B) Selection of the plasmid-free PXO99AΔxopN strains on NB plates with/without spectinomycin after pHM1B3-VD transformation. Spe-, without spectinomycin; Spe+, with spectinomycin added. (C) Representative agarose image for the presence/absence of pHM1 plasmid in the PXO99AΔxopN strain. Colony PCR was performed with pHM1-specific primers and negative PCR result indicated that the colonies were plasmid-free. M, 5 kb DNA ladder; Ctl, control. (D) Time course characterization of the pHM1B3-VD plasmid-curing system in PXO99AΔxopN cells bearing pHM1B4-xopN-crRNA. The percentage of plasmid-free cells was calculated at induction time 1, 2, 4, 6, 8, 10, 12 h after treatment of aTc in different dosages (200–3,000 ng/ml). (TIF) [file ppat.1010961.s002.tif]

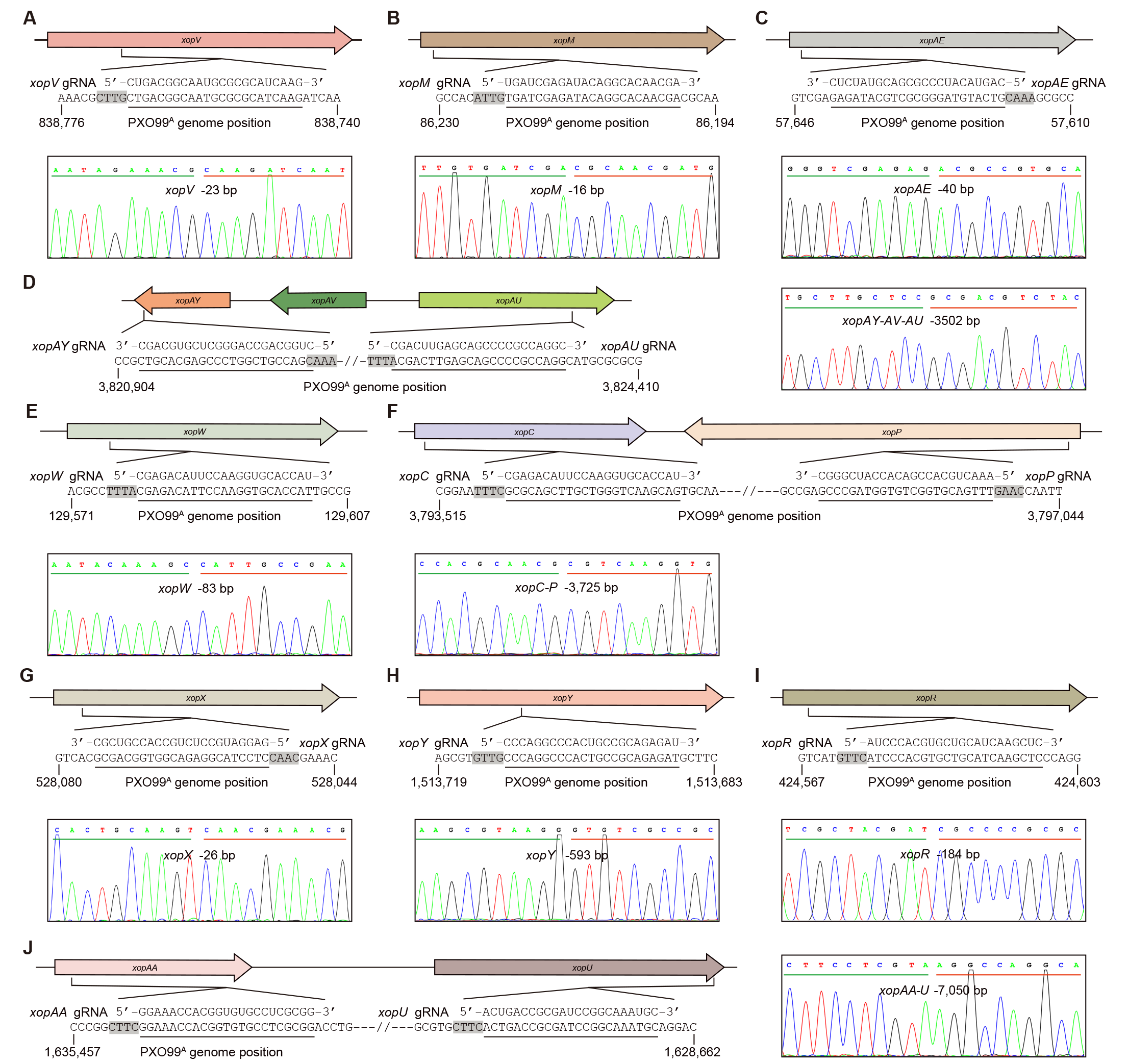

Supplement: S3 Fig — Specific gRNAs were designed to target xopV (A), xopM (B), xopAE (C), the xopAY-AV-AU gene cluster (D), xopW (E), the xopC-P gene cluster (F), xopX (G), xopY (H), xopR (I), and the xopAA-U gene cluster (J), and the CRISPR/FnCas12a-induced deletions are shown in Sanger sequencing chromatograms, respectively. The target regions in the PXO99A genome are underlined and the PAM sequences are marked by the black shadow. (TIF) [file ppat.1010961.s003.tif]

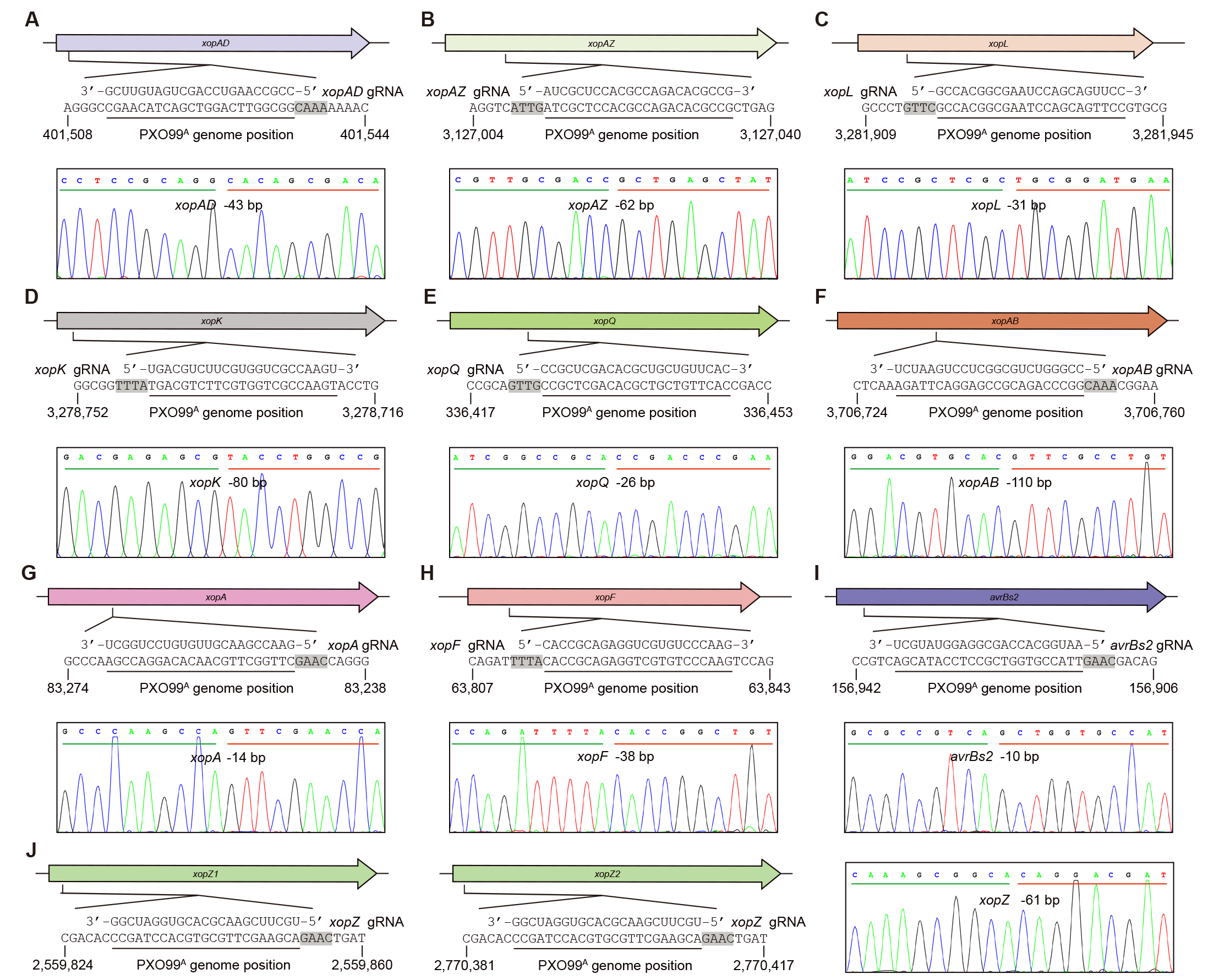

Supplement: S4 Fig — Specific gRNAs were designed to target xopAD (A), xopAZ (B), xopL (C), xopK (D), xopQ (E), xopAB (F), xopA (I), xopF (G), avrBs2 (H), and xopZ (J), and the CRISPR/FnCas12a-induced deletions are shown in Sanger sequencing chromatograms, respectively. The target regions in the PXO99A genome are underlined and the PAM sequences are marked by the black shadow. Two identical copies of xopZ have been annotated in the reference genome of PXO99A (NC_010717.2). (TIF) [file ppat.1010961.s004.tif]

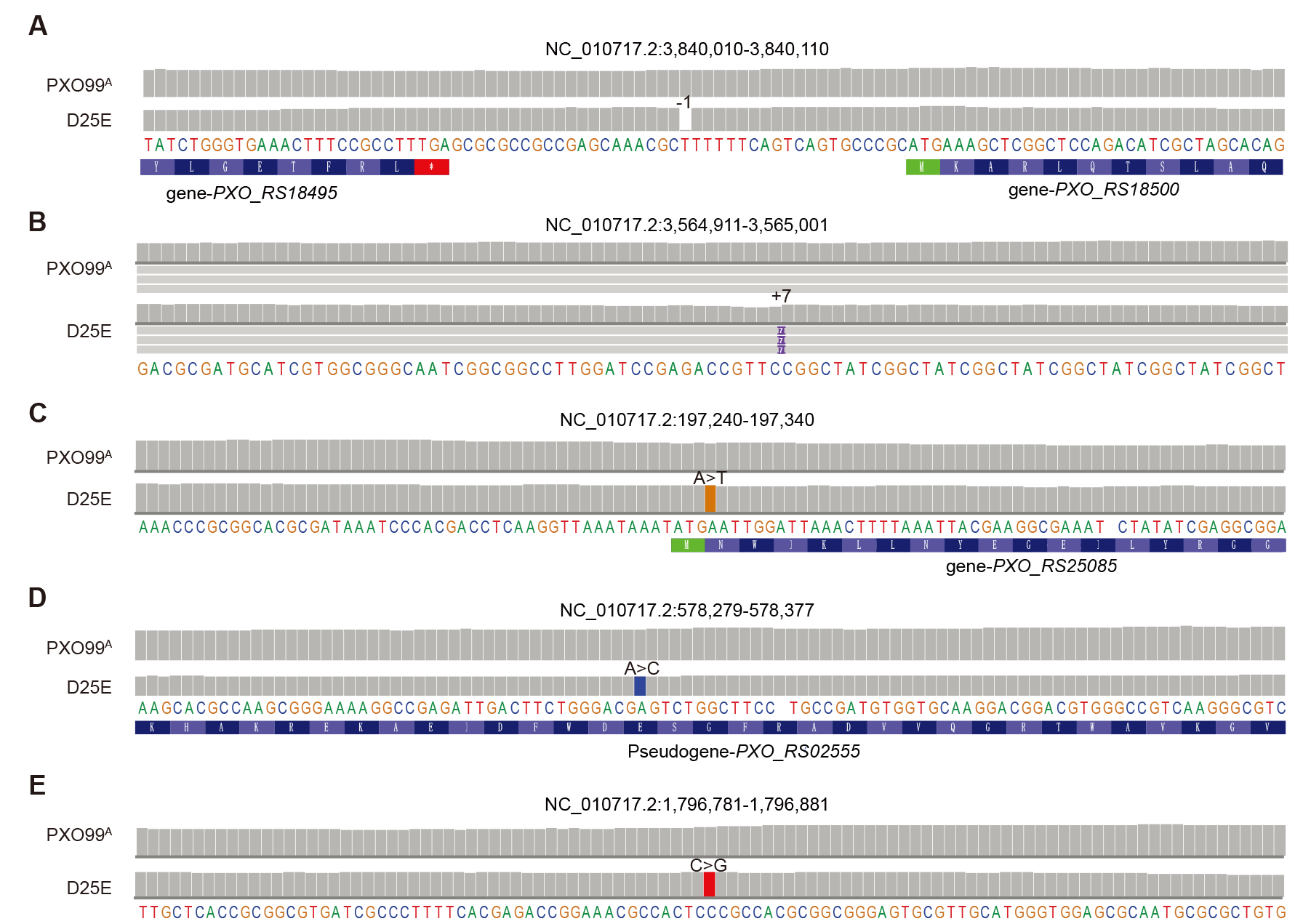

Supplement: S5 Fig — IGV genome browser views show 1-bp deletion (A), 7-bp insertion (B), and A-to-G (C), A-to-C (D), and C-to-G (E) nucleotide substitutions detected at 5 loci in the PXO99AD25E strain. (TIF) [file ppat.1010961.s005.tif]
